# Field-resolved high-order sub-cycle nonlinearities in a terahertz semiconductor laser

**DOI:** 10.1038/s41377-021-00685-5

**Published:** 2021-12-20

**Authors:** J. Riepl, J. Raab, P. Abajyan, H. Nong, J. R. Freeman, L. H. Li, E. H. Linfield, A. G. Davies, A. Wacker, T. Albes, C. Jirauschek, C. Lange, S. S. Dhillon, R. Huber

**Affiliations:** 1 Department of Physics, University of Regensburg, Regensburg, Germany; 2 Laboratoire de Physique de l’Ecole Normale Supérieure, ENS, Université PSL, CNRS, Sorbonne Université, Université de Paris, Paris, France; 3 School of Electronic and Electrical Engineering, University of Leeds, Woodhouse Lane, Leeds, UK; 4 Mathematical Physics and NanoLund, Lund University, Lund, Sweden; 5 Department of Electrical and Computer Engineering, Technical University of Munich, Munich, Germany; 6 Department of Physics, TU Dortmund University, Dortmund, Germany

**Keywords:** Quantum cascade lasers, Photonic devices

## Abstract

The exploitation of ultrafast electron dynamics in quantum cascade lasers (QCLs) holds enormous potential for intense, compact mode-locked terahertz (THz) sources, squeezed THz light, frequency mixers, and comb-based metrology systems. Yet the important sub-cycle dynamics have been notoriously difficult to access in operational THz QCLs. Here, we employ high-field THz pulses to perform the first ultrafast two-dimensional spectroscopy of a free-running THz QCL. Strong incoherent and coherent nonlinearities up to eight-wave mixing are detected below and above the laser threshold. These data not only reveal extremely short gain recovery times of 2 ps at the laser threshold, they also reflect the nonlinear polarization dynamics of the QCL laser transition for the first time, where we quantify the corresponding dephasing times between 0.9 and 1.5 ps with increasing bias currents. A density-matrix approach reproducing the emergence of all nonlinearities and their ultrafast evolution, simultaneously, allows us to map the coherently induced trajectory of the Bloch vector. The observed high-order multi-wave mixing nonlinearities benefit from resonant enhancement in the absence of absorption losses and bear potential for a number of future applications, ranging from efficient intracavity frequency conversion, mode proliferation to passive mode locking.

## Abstract (web summary)

Two-dimensional terahertz strong-field spectroscopy reveals wave-mixing processes up to eighth order in a free-running quantum cascade laser, unraveling its sub-cycle gain dynamics and nonlinearities in a regime of negative absorption.

## Graphical abstract

**Figure:**
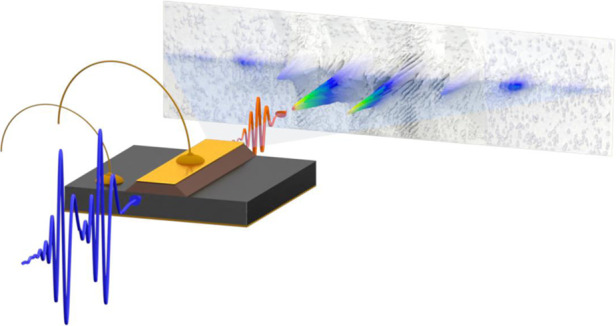
Two-dimensional terahertz strong-field spectroscopy reveals wave-mixing processes up to eighth order in a free-running quantum cascade laser, unraveling its sub-cycle gain dynamics and nonlinearities in a regime of negative absorption.

## Introduction

The terahertz (THz) window of the electromagnetic spectrum is at the heart of an ongoing revolution in optical sciences and technology. Ultrashort THz pulses have enabled resonant access to a broad variety of low-energy elementary excitations in condensed matter, such as plasmons, phonons, magnons, and Higgs modes in superconductors^1–7^. Strong THz fields have paved the way to extreme nonlinearities^2,8–11^ such as multi-wave mixing^12–15^, high-harmonic generation^9,16^, lightwave electronics^8^, and ultrafast nanoscopy^10,11,17,18^. Moreover, a wide range of novel photonics applications has employed THz radiation including high-resolution spectroscopy^1,19^, near-field microscopy^20^, and telecommunication concepts^21^. Thus, powerful, efficient, and compact sources of THz pulses and broadband frequency combs are in high demand, for which THz quantum cascade lasers (QCLs)^22^ have presented themselves as a particularly promising solution. These electrically pumped semiconductor lasers based on intersubband transitions and resonant tunneling in quantum wells have undergone considerable development over the last two decades. Important milestones include high-temperature^23,24^, high-power^25^, tunable^26^, and frequency comb operation^27,28^, as well as short pulse generation^29–31^.

In order to meet the growing demands of nonlinear optics and ultrashort pulse generation and to explore novel application fields, a detailed understanding and control of coherent and incoherent electron dynamics in operational THz QCLs is indispensable. In seminal pump-probe experiments^32–36^, gain relaxation oscillations and recovery times ranging from 0.2 ps to a few 10s ps have been reported for mid-infrared (MIR) QCLs. Also, four-wave mixing has been observed in time-integrated experiments^37^. In THz pump-probe measurements using weak pulses, incoherent gain dynamics of THz QCLs^38–40^ have been studied, and gain recovery times varying from 5 to 50 ps have been found. A recent two-dimensional (2D) spectroscopy approach^41^ based on injection seeding and RF switching (i.e., not free running) has yielded a gain recovery time of ~10 ps as well as the emergence of different nonlinear optical processes. Finally, 2D spectroscopy using strong THz pulses^42^ has allowed the investigation of incoherent absorption dynamics in an unbiased quantum cascade structure without a resonator.

Here we combine strong THz fields with a QCL, and show the first field-resolved 2D spectroscopy of a free-running THz QCL. Phase-stable, single-cycle THz transients with kV cm^−1^ peak fields strongly saturate the QCL gain by stimulated emission on ultrashort time scales, allowing for the direct observation of the gain recovery dynamics over the entire current operating range with a sub-cycle resolution by a subsequent THz pulse. Remarkably, we record coherent nonlinearities up to eight-wave mixing, which qualifies the QCL as an efficient nonlinear optical medium and enables a direct extraction of the dephasing time as a function of the bias current. This situation represents a rare scenario where resonantly enhanced high-order nonlinearities occur in a system with population inversion, bypassing the otherwise inevitable absorption losses. A density-matrix theory reproduces both incoherent and coherent multi-wave mixing signals and maps the internal QCL dynamics to the trajectory of the corresponding Bloch vector^43^. This approach may be particularly helpful in tailoring lasers for efficient intracavity frequency conversion, mode proliferation, and self-starting mode locking.

## Results

**Fig. 1:**
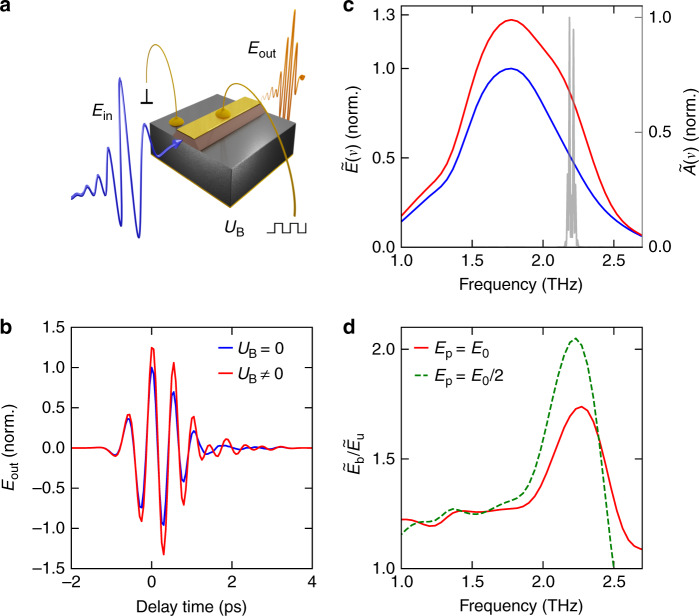
High-field spectroscopy of a THz QCL. **a** Experimental arrangement; gray: GaAs substrate, red: active medium, gold: waveguide and electrical contacts. The THz waveform *E*_in_ (blue) is focused onto the QCL facet and the transmitted waveform (*E*_out_, orange) is detected electro-optically. The rectangular modulation of the bias voltage allows the current-induced change of the nonlinear response to be extracted. **b** Measured THz waveform as a function of delay time *t*, after the transmission through the QCL with (red) and without (blue) bias current. **c** Amplitude spectrum $\tilde E\left( \nu \right)$ of the waveforms in **b** and lasing spectrum $\tilde A\left( \nu \right)$ of the QCL (gray). **d** Modulation of the transmission through the QCL as a function of frequency, defined as the ratio of the transmission spectra of the biased and the unbiased QCL (see **c**) for two peak electric fields *E*_p_ = *E*_0_ = 1.4 kV cm^−^^1^ (red) and *E*_p_ = *E*_0_/2 (green, broken curve)

Our QCL is based on an Al_0.1_Ga_0.9_As/GaAs heterostructure (described in detail elsewhere^44^) that shows laser action at 2.2 THz above a threshold bias current of *I*_th_ = 910 mA. The laser structure was processed, by wet-chemical etching, into a laser ridge, 250 µm wide and with a thickness of 200 µm, which includes both the active region and the substrate (Fig. 1a). Contact electrodes were deposited on the top and the sides of the laser ridge for current injection. The sample was cleaved into a laser resonator of length 2.9 mm, soldered onto a gold-coated copper base, and finally mounted on the cold finger of a liquid-helium cryostat. The bias voltage provided by an external power supply was modulated at a frequency of 500 Hz and synchronized with the THz probe pulses, allowing for a direct extraction of the bias-induced change in transmission. Single-cycle THz pulses with a tunable peak field strength of up to 3 kV cm^−1^ and a repetition rate of 1 MHz generated by tilted-pulse-front optical rectification of intense near-infrared (NIR) laser pulses in lithium niobate (see “Materials and methods” section) are polarized parallel to the growth direction of the heterostructure and coupled into the QCL waveguide through the front facet (Fig. 1a). The THz transient propagates through the active medium and is partially transmitted through the end facet of the QCL. We fully resolve the amplitude and the absolute phase of the transmitted waveform by electro-optic sampling in a GaP crystal, as a function of the delay time *t*. This field-resolved detection allows us to distinguish unequivocally the transmitted THz fields, which have a fixed phase relation with the incident pulses, from the steady-state THz radiation emitted by the free-running QCL. This way, we can study nonlinearities both below and above the laser threshold with excellent contrast despite strong QCL emission.

Figure 1b displays the transmitted THz waveform after a single pass through the structure for the biased (red) and the unbiased QCL (blue). The field transmitted through the biased laser is ~30% higher and shows a clear oscillation for delay times *t* > 1 ps owing to the amplification of the incident THz pulse by stimulated emission in the inverted active medium. The Fourier transform of the two waveforms (Fig. 1c) provides the spectral characteristics of the QCL gain. At a frequency of 2.2 THz, the spectral amplitude of the biased device, $\tilde E$_b_(ν), features an increase over the unbiased situation, $\tilde E$_u_(ν). This frequency agrees very well with the designed lasing spectrum of the QCL (Fig. 1c). The overall redistribution of charge carriers inside the heterostructure upon biasing leads to a broadband background of increased transmission^34^.

For a more quantitative analysis of the gain, Fig. 1d shows the ratio of transmission, $\tilde E$_b_(ν)/$\tilde E$_u_(ν) (red curve), measured with an incident THz peak field of 1.4 kV cm^−1^ (see Supplementary Information for the corresponding phase spectrum). The dominant gain maximum in the QCL active medium at a frequency of 2.2 THz reaches values of up to 37% above the baseline. The latter exceeds unity because of the overall enhancement of transmission by electrical pumping. Assuming a spatially independent linear gain over the cavity length, we obtain a gain coefficient of $\alpha _{E_0} \approx 2.2\,{{{\mathrm{cm}}}}^{ - 1}$. Interestingly, the gain spectrum depends sensitively on the incident THz field strength. A transmission spectrum measured at half the peak incident electric field of the THz pulse shows a similar base level (Fig. 1d, green curve), yet the resonant gain ($\alpha _{E_0/2} \approx 3.4\,{{{\mathrm{cm}}}}^{ - 1}$) reaches values of up to 64% above the baseline—almost twice the value of the high-field case. This drastic change attests to a strong saturation of the QCL gain^39^. Moreover, the linewidth increases from 0.35 to 0.39 THz as the incident THz field doubles, which we attribute to power broadening and a reduced lifetime of the upper laser state under intense excitation. Inhomogeneous broadening effects play only a minor role, since the relative variation of the well thickness of order 1% ^45^ corresponds to a broadening of the laser transition of less than ~40 GHz. In this strongly nonlinear regime, the overall response may result from a variety of simultaneous competing or cooperating nonlinear optical elementary processes. Yet, their individual contributions, which could provide key insights into the microscopic electron dynamics in principle, remain hidden in single-pulse transmission studies.

**Fig. 2:**
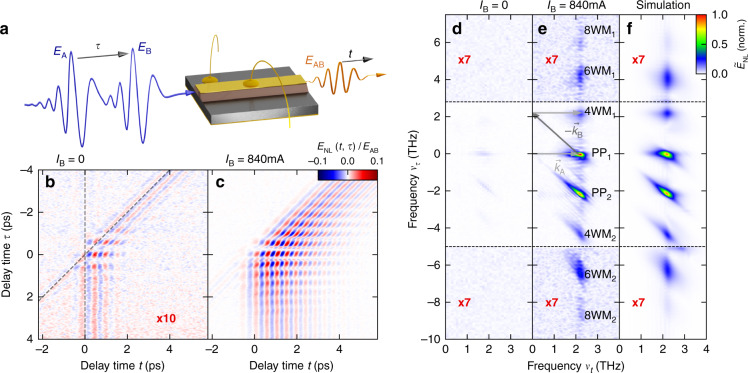
Field-resolved 2D THz spectroscopy of a free-running QCL. **a** Experimental arrangement. The THz-waveforms *E*_A_ and *E*_B_ (blue) are focused onto the QCL facet and the transmitted waveform (*E*_AB_, orange) is detected electro-optically. **b** Electric field emitted by the nonlinear polarization, *E*_NL_(*t*, *τ*), of the unbiased QCL as a function of the delay times *t* and *τ*. The electric field is multiplied by a factor of 10 and color-coded as in **c**. **c** *E*_NL_(*t, τ*), as in **b**, but for biased QCL with a current of *I*_B_ = 840 mA. **d** 2D-amplitude spectrum $\tilde E_{{{{\mathrm{NL}}}}}\left( {\nu _t,\nu _\tau } \right)$ of the nonlinear response (*I*_B_ = 0) in **b**. The spectrum is multiplied by a factor of 7 for *ν*_τ_ < −5 THz and *ν*_τ_ > 2.8 THz and color-coded as in **e**. **e** 2D-amplitude spectrum $\tilde E_{{{{\mathrm{NL}}}}}\left( {\nu _t,\nu _\tau } \right)$ of the nonlinear response (*I*_B_ = 840 mA) in **c**. For better visibility of the 6WM and 8WM signals, the corresponding part of the spectrum is multiplied by a factor of 7. **f** Corresponding simulated 2D-amplitude spectrum $\tilde E_{{{{\mathrm{NL}}}}}\left( {\nu _t,\nu _\tau } \right)$

Field-resolved two-dimensional THz spectroscopy, in contrast, represents a powerful strategy to disentangle all relevant coherent and incoherent nonlinear processes^12,14,46,47^ (see “Materials and methods” section). To this end, two identical THz waveforms *E*_A_(*t*) and *E*_B_(*t*, *τ*), which are mutually delayed by a variable time *τ*, are focused onto the QCL (Fig. 2a). The first pulse induces a polarization or population change inside the active medium, which is subsequently interrogated by the second pulse. The total transmitted field *E*_AB_(*t*, *τ*) is recorded with absolute phase and amplitude using electro-optic sampling and consists of a linear superposition of the incident fields and the nonlinear contributions, *E*_NL_(*t*, *τ*). Synchronous mechanical chopping of the two incident THz beams *E*_A_(*t*) and *E*_B_(*t*, *τ*) isolates their individual contributions, and by subtracting them from the transmitted signal *E*_AB_(*t*, *τ*), we are left with the nonlinear signal *E*_NL_(*t*, *τ*) = *E*_AB_(*t*, *τ*) – *E*_A_(*t*) – *E*_B_(*t*, *τ*).

Figure 2b shows the resulting 2D field map *E*_NL_(*t*, *τ*) for the unbiased QCL as a function of the delay time *t* of the electro-optic sampling process and the delay time between the two pulses, *τ*. The strongest nonlinearities occur near *τ* = 0 ps, where both pulses overlap temporally. By varying *τ*, the total incident field is strongly modulated leading to a pronounced variation of *E*_NL_. Even for delay times |*τ*| > 2 ps and *t* > 0 ps, where the two THz pulses no longer overlap in time, nonlinear optical signals are observed. On the electro-optic time axis, *t*, they set in with the second pulse, which is pulse A or pulse B (Fig. 2b, broken lines) depending on *τ*. The nonlinear response manifests as oscillations at the transition frequency of the unbiased QCL at $\nu _{I = 0}$ = 1.7 THz (Fig. 2d and Supplementary Information). In sharp contrast, passing a bias current of 840 mA, slightly below the laser threshold, through the QCL leads to a qualitatively different response. First, *E*_NL_ increases by more than one order of magnitude, up to almost 10% of the amplitude of the transmitted THz pulses, *E*_AB_ (Fig. 2c). Second, the coherent modulation following the second pulse is more long-lived and blue shifted to the laser resonance of *ν*_L_ = 2.2 THz. Third, the modulation of *E*_NL_ along *t*, observed only near time zero for the unbiased structure, now persists for much larger delays |*τ*|, signifying the presence of coherent nonlinear processes.

To first order, the observed nonlinear signal may be understood considering the saturation of the active QCL gain discussed above. The first THz pulse to arrive at the structure depletes the population of the upper laser level by stimulated emission, decreasing the transmission of the second pulse through the active medium within the gain recovery time. As |*τ*| increases, the gain is restored by the pump current. Moreover, the first THz pulse imprints its phase on the oscillating intersubband polarization. The relative phase of Δφ = *τν*_L_ between the induced coherent polarization and the second pulse determines whether the latter can further reduce or restore the population inversion by stimulated emission or absorption.

To expand *E*_NL_ systematically into its constituent nonlinear processes, we perform a 2D Fourier transform of the time-domain data of Fig. 2c. By comparing the resulting amplitude spectrum $\tilde E$_NL_ (Fig. 2e) with the spectrum of the unbiased case (Fig. 2d), we observe that the nonlinearities do not only drastically increase, but also higher-order multi-wave mixing signals appear. The distinct maxima of $\tilde E$_NL_ are located at (*ν*_*t*_, *ν*_*τ*_) = (*ν*_L_, *n* × *ν*_L_), where *n* is an integer number. Given the time order of the 2D scans, the linear polarization of the incident waves A and B resonant with the laser transition would be located at (*ν*_L_, 0), and (*ν*_L_, −*ν*_L_), respectively. Starting from the origin (0,0), every maximum of $\tilde E$_NL_ can be navigated by adding or subtracting a suitable number of these two vectors representing photons from pulses A and B (see example path in Fig. 2e). This Liouville path analysis allows us to assign uniquely the number and the phase of the interacting photons contributing to the nonlinear optical process that gives rise to a specific maximum^12^. The dominant structures at (*ν*_L_, 0) and (*ν*_L_, −*ν*_L_) are incoherent pump-probe (PP_1_ and PP_2_) contributions^12,14,46,47^, for which the phase of either pulse A (PP_2_) or pulse B (PP_1_) is irrelevant (see “Materials and methods” section for details). Therefore, they are directly linked to population dynamics emerging from the modulation of the QCL gain by saturation and pump-induced restoration of the population inversion. In contrast, the signatures at (ν_L_, ν_L_), and (ν_L_, −2ν_L_) are caused by four-wave mixing (4WM_1_ and 4WM_2_) processes originating from purely coherent nonlinear polarization dynamics which depend on the phase of all participating photons (see “Materials and methods” section for details). The relative strength of the four-wave mixing signals amounts to as much as ~1% of the amplitude of the transmitted THz pulses, *E*_AB_, indicating an unusually large third-order susceptibility of at least 10^10^ pm^2^ V^−^^2^ (see Supplementary Information for details). Remarkably, the QCL’s strongly nonlinear response also includes six- and even eight-wave-mixing signals at (*ν*_L_, −3*ν*_L_) and (ν_L_, −4*ν*_L_), respectively, corresponding to the interaction of as many as eight photons from both THz pulses. To the best of our knowledge, these observations mark the highest-order nonlinearity reported for QCLs so far.

**Fig. 3:**
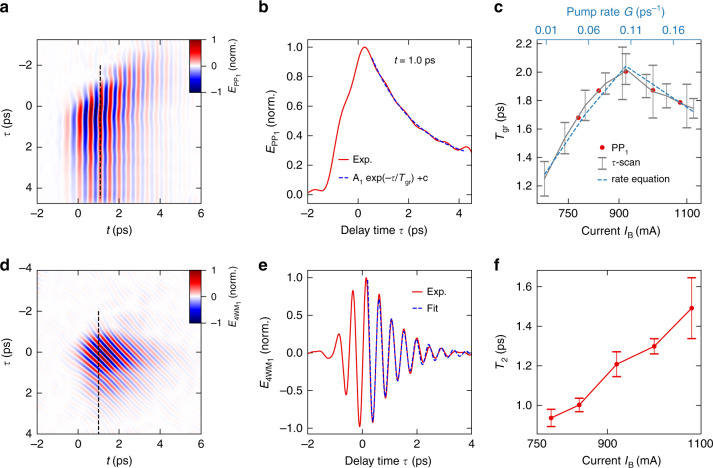
Quantitative analysis of the coherent and incoherent dynamics in the QCL. **a** Back-transformed electric field corresponding to the pump-probe signal $E_{{{{\mathrm{PP}}}}_1}$. **b** Slice through $E_{{{{\mathrm{PP}}}}_1}$(*t*, *τ*) at a delay time *t* = 1.0 ps (see black dashed line in **a**). Blue dashed line: numerical fit of function *A*_1_ exp(−*τ*⁄*T*_gr_)+*C*. **c** Gain recovery time *T*_gr_ of the QCL as a function of the bias current, extracted from the PP_1_ signal (red dots) and the full nonlinear time domain signal (gray). The error bars display the uncertainty of the numerical fit. Light blue dashed line: *T*_gr_ as a function of the pump rate *G*, extracted from the rate equation model. **d** Back-transformed electric field corresponding to the four-wave mixing signal $E_{4{{{\mathrm{WM}}}}_1}$. **e** Slice through $E_{4{{{\mathrm{WM}}}}_1}$(*t*, *τ*) at an example delay time *t* = 1.0 ps (see black dashed line in **d**). Blue dashed line: numerical fit of function $A_2\sin \left( {2\pi \nu_{{{\mathrm{L}}}}\tau + \phi} \right)\exp \left( { - \frac{\tau }{{T_2}}} \right)$. **f** Decay time of the coherent population *T*_2_ as a function of the bias current. The error displays the uncertainty of *T*_2_ for different delay times between 0.45 and 1.75 ps

This wealth of information enables us to extract valuable parameters of the microscopic electron dynamics in the free-running QCL. Since the nonlinearities studied here occur within a single pass through the QCL cavity, propagation effects do not play a dominant role and spatially averaged dynamics provide a meaningful description (Supplementary Information). As a first test, we quantitatively retrieve the gain recovery time, *T*_gr_, for a series of values of *I*_B_ from below to above the laser threshold, which for THz QCLs has only been available for weak saturation to date^41^. For strong gain depletion, we proceed by selectively back-transforming the PP_1_ signal into the time domain (Fig. 3a). The exponential decay of the corresponding nonlinear field component along the *τ* axis takes the form

1 $$E_{{{{\mathrm{PP}}}}1}\left( {t = 1.0\,{{{\mathrm{ps}}}},\tau } \right) = A_1\exp \left( { - \frac{\tau }{{T_{{{{\mathrm{gr}}}}}}}} \right) + C$$

when evaluated at a fixed delay time, such as *t* = 1.0 ps (dotted black line in Fig. 3a). Here, *A*_1_ is the signal amplitude and *C* is an offset accounting for thermal effects. Alternatively, *T*_gr_ can be extracted from the measured time-domain signal *E*_NL_ directly since *E*_PP1_ is the only contribution that does not oscillate in the *τ* direction (Fig. 3b, see Supplementary Information for details). The excellent agreement of both approaches allows us to determine *T*_gr_ reliably as a function of the bias current, *I*_B_ (Fig. 3c), by rapidly measuring one-dimensional sets of *E*_NL_ for constant *t*. Starting at a value of *T*_gr_ = 1.3 ps, below the laser threshold (*I*_B_ = 680 mA), *T*_gr_ grows with increasing *I*_B_ until it reaches a maximum of (2.0 ± 0.2) ps at the laser threshold current of *I*_th_ = 910 mA, followed by a drop upon further increase of *I*_B_. The overall time constants are remarkably short given that the laser emission occurs far below the optical phonon energy and the lattice temperature is kept below 10 K. Microscopically, the characteristic current dependence of *T*_gr_ can be understood within a rate equation model that goes beyond the picture of a pure quantum cascade amplifier by considering the electrically pumped laser transition in a two-level picture and the feedback by the laser cavity (see “Materials and methods” section). Below the threshold, increasing pump currents boost the population inversion *w* and hence the losses by amplified spontaneous emission, such that the recovery of *w* takes longer. When *I*_B_ reaches the laser threshold, gain clamping limits a further increase of *w* and *T*_gr_ is maximal. With yet higher pump currents, *T*_gr_ finally drops because of faster repopulation of the upper laser level, as observed in Fig. 3c. Although longer relaxation times may still exist owing to the complex transport in a QCL band structure, the predominant gain relaxation time in our experiment is extremely fast (*T*_gr_ ≤ 2 ps).

Going beyond population dynamics, we can also analyze the nonlinear polarization of the free-running THz QCL for the first time. By back-transforming the four-wave mixing signal 4WM_1_ (Fig. 3d) and fitting the experimental data at a fixed delay time (e.g., *t* = 1.0 ps) with the exponentially decaying oscillatory function

2 $$E_{4{{{\mathrm{WM}}}}1}\left( {t = 1.0\,{{{\mathrm{ps}}}},\tau } \right) = A_2\sin \left( {2\pi \nu _{{{\mathrm{L}}}}\tau + \phi } \right)\exp \left( { - \frac{\tau }{{T_2}}} \right)$$

we extract the polarization decay time, *T*_2_. Here, *A*_2_ is the signal amplitude and *ϕ* is a constant phase. Given the excellent agreement of the above fit function with the experimental data (Fig. 3e), faithful values of the decay time of the nonlinear polarization can be extracted for pump currents below and above the threshold. Interestingly, *T*_2_ monotonically increases from *T*_2_ = 0.9 ps at *I*_B_ = 780 mA by a factor of almost 1.6 to *T*_2_ = 1.5 ps at *I*_B_ = 1080 mA (Fig. 3f).

## Discussion

Although analyzing selected nonlinear optical processes provides access to key information associated with the population and polarization dynamics, our access to a large number of different high-order nonlinearities (Fig. 2e) facilitates a much more comprehensive insight into the microscopic sub-cycle dynamics. As a first demonstration, we utilize the optical Bloch equations to track the full THz-induced coherent trajectory of the Bloch vector of the resonant laser transition, and explain the emergence of the various nonlinear optical processes all at once. The gain medium is described by a density matrix *ρ* coupled to the self-consistently calculated THz cavity electric field, *E*(*t*), leading to a Hamiltonian of the form:

3 $$H = \left( {\begin{array}{*{20}{c}} 0 & {\mu _{12}E\left( t \right)} \\ {\mu _{12}E\left( t \right)} & {h\nu _{{{\mathrm{L}}}}} \end{array}} \right)$$

Here, *μ*_12_ and *ν*_L_ represent the dipole moment and the frequency of the laser transition, respectively. The dynamics are calculated beyond the rotating-wave approximation by solving the von Neumann equation. The equations of motion for the density matrix elements are given as:

4a $$\dot \rho_{11} = \frac{i}{\hbar }\mu _{12}E\left( t \right)\left( {\rho_{12} - \rho_{12}^\ast } \right) - \frac{1}{{T_{{{{\mathrm{gr}}}}}}}\left( {\rho _{11} - \rho _{11}^0} \right)$$

4b $$\dot \rho _{22} = \frac{i}{\hbar }\mu _{12}E\left( t \right)\left( {\rho _{12}^ \ast - \rho _{12}} \right) - \frac{1}{{T_{{{{\mathrm{gr}}}}}}}\left( {\rho _{22} - \rho _{22}^0} \right)$$

4c $$\dot \rho _{12} = \frac{i}{\hbar }\mu _{12}E\left( t \right)\left( {\rho _{11} - \rho _{22}} \right) - i2\pi \nu _{{{\mathrm{L}}}}\rho _{12} - \frac{{\rho _{12}}}{{T_2}} = \dot \rho _{21}^ \ast$$

In the stationary state without THz excitation, we assume the QCL to exhibit a slight population inversion $w_0 = \rho _{22}^0 - \rho _{11}^0$. The electric field *E*(*t*) is calculated by considering the incident THz field, *E*_THz_, as well as the electric field reradiated by the electronic polarization, *ρ*_12_:

5 $$E\left( t \right) = E_{{{{\mathrm{THz}}}}} - \Gamma \mu _0\frac{c}{{2n}}\dot \rho _{12}$$

where *μ*_0_ denotes the vacuum permeability, *c* is the speed of light, and *n* is the refractive index of the material. Γ is a constant accounting for the coupling of the electron ensemble to the cavity field (Supplementary Information). All parameters are taken from the literature or auxiliary measurements as far as available. Only *T*_1_ and *T*_2_ are used as fitting parameters. Analogously to the experiment, we derive the nonlinear field *E*_NL_ from calculations of the individual configurations including both or only one of the THz pulses. The resulting 2D-amplitude spectrum (Fig. 2f) reproduces the emergence of all nonlinear signals including pump-probe, four-, six-, and eight-wave-mixing signals. Furthermore, the simulation reveals the underlying temporal evolution of the microscopic QCL dynamics including the population inversion *w* = *ρ*_22_ – *ρ*_11_ and the polarization *ρ*_12_ = *u* + *iv* as a function of *t* and *τ*.

**Fig. 4:**
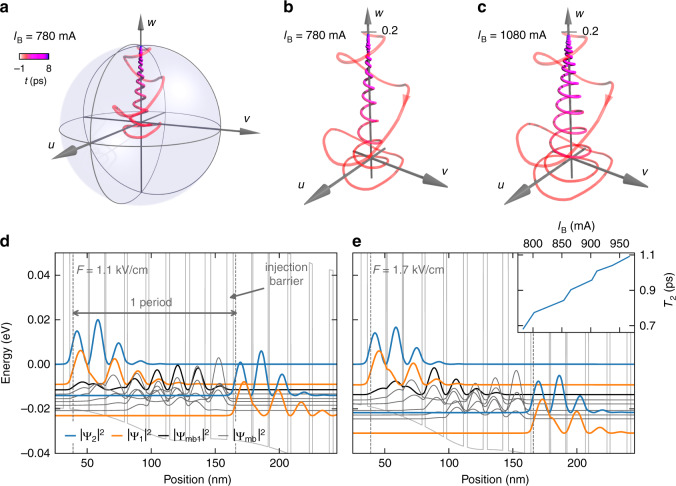
Microscopic picture of the QCL dynamics. **a** Temporal evolution of the QCL’s density matrix, shown in a reduced Bloch sphere with a radius of 0.2 for a delay time of *τ* = 0 ps and a bias current of *I*_B_ = 780 mA. **b** Close-up view of the trajectory of the Bloch vector shown in **a**. **c** As **b**, but for *I*_B_ = 1080 mA. **d** Simulated band diagram of the QCL under an electric field of *F* = 1.1 kV cm^−1^. The blue and orange lines represent the squared modulus of the wave function of the upper and lower laser level (|Ψ_2_|^2^ and |Ψ_1_|^2^, respectively). Additionally, the highest miniband state |Ψ_mb1_|^2^ as well as the other miniband states |Ψ_mb_|^2^ are shown in black and gray. **e** As in **d** but for a bias voltage of *F* = 1.7 kV cm^−^^1^. Inset: Calculated dependence of *T*_2_ on the bias current *I*_B_

Figure 4a shows the evolution of the QCL’s state in a reduced Bloch sphere representation for *τ* = 0 ps. Unlike in an equilibrium two-level system, the initial state of the Bloch vector is not given by the south pole of the Bloch sphere, but an incoherent state (*u* = *v* = 0) located slightly above *w* = 0 corresponding to a moderate population inversion of the gain medium, prepared by the pump current. The intense THz field drives the Bloch vector onto a coherent trajectory, which, unlike in a conventional equilibrium two-level system, points downwards as the pump pulse coherently depletes the population inversion by stimulated emission within a single cycle of the THz carrier wave. Subsequently, electrical pumping restores the population inversion while the free induction decay of the induced polarization causes a spiraling trajectory back to the stationary incoherent starting point. Because the THz field is resonant to the laser transition, these dynamics and all multi-wave mixing nonlinearities associated with them are resonantly enhanced. Yet, resonant enhancement in a running laser is associated with gain rather than absorption, making QCLs a highly attractive nonlinear optical medium.

Importantly, the coherent Bloch trajectory sensitively depends on the bias current. The close-up in Fig. 4b highlights the dynamics for a low current of *I*_B_ = 780 mA, where the coherent polarization given by the excursion in the *u* and *v* directions of the Bloch trajectory rapidly collapses. In contrast, for a bias current of *I*_B_ = 1080 mA, which is above threshold (Fig. 4c), the polarization maintains a much larger amplitude until the population inversion is fully restored. To establish a solid microscopic understanding of these bias-dependent coherent dynamics, we simulated the underlying electronic transport with a density matrix ensemble Monte Carlo approach coupled to a Schrödinger–Poisson solver. This allows us to extract the relevant scattering, tunneling, and dephasing rates self-consistently. We accounted for eight states per QCL period.

Figure 4d, e displays the resulting band diagram of the QCL under two typical bias fields of 1.1 kV cm^−^^1^ and 1.7 kV cm^−1^ together with the computed dephasing time, *T*_2_, of the lasing transition (Fig. 4e, inset). The theory clearly confirms a monotonic rise of *T*_2_ with *I*_B_ as observed experimentally (Fig. 3f). In our simulations, this behavior is mainly caused by the decreasing overlap of the lower laser level with other levels with increasing bias. As seen in Fig. 4d, e, a stronger bias induces a more pronounced energy spread of the minibands, which reduces the rate of electron–electron scattering between them and limits scattering out of the lower laser level. This behavior is supported by the bias-dependent spatial overlap of the miniband states. For a low bias field of 1.1 kV cm^−^^1^ (Fig. 4d), the squared wave functions of the miniband states (e.g., |Ψ_mb1_|^2^, black curve) are delocalized over almost the full injector period whereas they spatially separate with increasing bias fields (1.7 kV cm^−1^, Fig. 4e), suppressing phase destroying scattering events in the miniband states. Since our model does not explicitly consider back action by the laser cavity, we restricted our simulations to currents below the threshold. The new 2D spectroscopy data hence provide an important benchmark for future quantum theories treating both electronic transport processes and intracavity light-matter interaction on equal sub-cycle footing.

In conclusion, the first 2D spectroscopy of a free-running THz QCL revealed strong nonlinearities including up to eight-wave mixing, on a sub-cycle time scale. Besides extremely fast gain recovery times down to 1.2 ps, our approach reveals the coherent trajectory of the Bloch vector itself, unlocking a previously inaccessible dimension of electron dynamics in QCLs. While our experiments were carried out with a prototypical design of a THz QCL, we expect this new comprehensive access to coherent and incoherent response functions to be broadly applicable to all kinds of innovative device architectures. In particular, mode-locked THz QCLs will depend on an in-depth understanding of the carrier-wave dynamics of the gain, the coherent polarization, and electronic transport for dispersion management and single-cycle pulse formation. Moreover, highly efficient and tunable multi-wave mixing opens up exciting perspectives for intracavity frequency conversion, multiplexing, potentially even quantum squeezing, and strong-field light-matter coupling in a single compact electrically pumped device.

## Materials and methods

### Experimental setup

Phase-locked THz pulses are generated by optical rectification of near-infrared laser pulses of a duration of 220 fs from an Yb:KGW amplifier (repetition rate, 1 MHz) in a cryogenically cooled (*T* = 77 K) lithium niobate crystal in a tilted pulse-front configuration. The THz waveforms (Supplementary Fig. S1) feature peak fields of up to 3 kV cm^−1^ and a duration of 0.84 ps of the intensity envelope (FWHM), corresponding to 1.3 optical cycles of the carrier frequency of 1.6 THz (Supplementary Fig. S1, inset). A Michelson interferometer with a silicon beamsplitter creates two identical copies of the THz pulses which are subsequently delayed with respect to each other by a variable delay time *τ*, before being recombined collinearly. The pulse pair is coupled into the facet of the QCL within the cryostat and the transmitted THz radiation is focused onto a GaP crystal of a thickness of 1 mm for electro-optic detection, using a small portion of the laser pulse as a gate. In each of the two interferometer arms, a mechanical chopper is placed in an intermediate focus, enabling independent modulation of both THz pulse trains.

### 2D THz spectroscopy

We employ 2D THz spectroscopy to disentangle nonlinear processes inside the QCL by a Liouville path analysis which relates each nonlinear signature in the two-dimensional spectra to a specific wave vector combination of the incident fields^47^. For example, χ^(3)^ nonlinearities, such as pump-probe and four-wave mixing processes, combine three photons of the two incident fields. In case of the pump-probe signal PP_1_ at frequencies of (*v*_*t*_, *v*_τ_) = (*v*_L_, 0), a third-order polarization

6 $$P_{{{{\mathrm{PP}}}}_1}^{\left( 3 \right)}\left( {\nu_{{{\mathrm{L}}}}} \right) = \epsilon_0\chi ^{\left( 3 \right)}\left( {\nu_{{{\mathrm{L}}}},\nu_{{{\mathrm{L}}}}, - \nu_{{{\mathrm{L}}}}} \right)\tilde {\mathcal{E}}_{{{\mathrm{A}}}}\left( {\nu_{{{\mathrm{L}}}}} \right)\tilde {\mathcal{E}}_{{{\mathrm{B}}}}\left( {\nu_{{{\mathrm{L}}}}} \right)\tilde {\mathcal{E}}_{{{\mathrm{B}}}}^ \ast \left( { - \nu_{{{\mathrm{L}}}}} \right)$$

is created by one photon from the incident pulse A and two photons from pulse B, represented by the complex-valued spectral amplitudes $\tilde{\mathcal{E}}_{\mathrm{A}}$ and $\tilde{\mathcal{E}}_{\mathrm{B}}$, respectively. Since the field components $\tilde{\mathcal{E}}_{\mathrm{B}}({\nu_{\mathrm{L}}})$ and $\tilde{\mathcal{E}}_{\mathrm{B}}^\ast({-\nu_{\mathrm{L}}})$ are each other’s complex conjugate with mutually inverted phases (see inverted sign of their frequencies), the phase of field B cancels. Only the phase of the probe photon from pulse A remains imprinted in the nonlinear polarization $P_{{{{\mathrm{PP}}}}_1}^{\left( 3 \right)}$. Therefore, PP_1_ is incoherent with respect to the phase of pulse B, whereas PP_2_, where A and B switch roles, is incoherent with respect to the phase of pulse A. In contrast, four-wave mixing nonlinearities preserve the phases of both pulses. Considering the 4WM_1_ signal where (*v*_*t*_, *v*_τ_) = (*v*_L_, *v*_L_), two photons from pulse A and one photon from pulse B of inverted phase contribute to the resulting third-order polarization:

7 $$P_{4{{{\mathrm{WM}}}}_1}^{\left( 3 \right)}\left( {\nu_{{{\mathrm{L}}}}} \right) = \epsilon_0\chi ^{\left( 3 \right)}\left( {\nu_{{{\mathrm{L}}}},\nu_{{{\mathrm{L}}}}, - \nu_{{{\mathrm{L}}}}} \right)\tilde {\mathcal{E}}_{{{\mathrm{A}}}}\left( {\nu_{{{\mathrm{L}}}}} \right)\tilde {\mathcal{E}}_{{{\mathrm{A}}}}\left( {\nu_{{{\mathrm{L}}}}} \right)\tilde {\mathcal{E}}_{{{\mathrm{B}}}}^ \ast \left( { - \nu_{{{\mathrm{L}}}}} \right)$$

In conclusion, nonlinear pump-probe signals relate to the incoherent population dynamics of the system, while four-wave mixing or higher-order multi-wave mixing signals represent the coherent nonlinear polarization.

A conventional graphical representation of this Liouville path decomposition plots the contributing wave vectors in 2D frequency space, as shown in Fig. 2e. Here, each signature can be reached by a path consisting of an integer linear combination of the wave vectors **k**_A_ = (*v*_L_, 0) and **k**_B_ = (*v*_L_, −v_L_), each representing one photon from pulse A or B, respectively. Correspondingly, the signatures at **k**_PP1_ = (*v*_L_, 0) and **k**_PP2_ = (*v*_L_, −*v*_L_) belong to the Liouville paths **k**_PP1_ = **k**_A_ + **k**_B_ − **k**_B_ and **k**_PP2_ = **k**_A_ − **k**_A_ + **k**_B_, respectively, while four-wave mixing signals occurring at **k**_4WM1_ = (*v*_L_, *v*_L_) and **k**_4WM2_ = (*v*_L_, –2*v*_L_) are decomposed to **k**_4WM1_ = **k**_A_ + **k**_A_ − **k**_B_ and **k**_4WM2_ = **k**_B_ + **k**_B_ − **k**_A_.

### Rate equation model for the QCL gain dynamics

To model the gain recovery time of the QCL we assume a two-level system with an electron reservoir and a drain for the upper and lower level, respectively, located inside the QCL cavity. The electron number *N*_1_ and *N*_2_ in the lower and upper laser level as well as the photon number *N*_ph_ in the cavity are described by the following rate equations:

8 $$\frac{{{{{\mathrm{d}}}}N_1}}{{{{{\mathrm{d}}}}t}} = - \frac{{N_1}}{{\tau _1}} + \frac{{N_2}}{{\tau _2}} + B_{21}\left( {N_2 - N_1} \right) \cdot \rho _0N_{{{{\mathrm{ph}}}}}$$

9 $$\frac{{{{{\mathrm{d}}}}N_2}}{{{{{\mathrm{d}}}}t}} = \left( {N_\mathrm{e} - N_2} \right) \cdot G - \frac{{N_2}}{{\tau _2}} - B_{21}\left( {N_2 - N_1} \right) \cdot \rho _0N_{{{{\mathrm{ph}}}}}$$

10 $$\frac{{{{{\mathrm{d}}}}N_{{{{\mathrm{ph}}}}}}}{{{{{\mathrm{d}}}}t}} = - \frac{{N_{{{{\mathrm{ph}}}}}}}{{\tau _{{{{\mathrm{cav}}}}}}} + B_{21}\left( {N_2 - N_1} \right) \cdot \rho _0N_{{{{\mathrm{ph}}}}} + \beta \cdot \delta \left( {t - t_{{{{\mathrm{pert}}}}}} \right)$$

Here, *τ*_1_ and *τ*_2_ describe the lifetimes of the lower and upper laser level. The losses of the cavity are considered by a corresponding cavity lifetime, *τ*_cav_. To fit the experimental data and in general agreement with literature values^36,48,49^, we choose *τ*_1_ = 4.3 ps, *τ*_2_ = 5.5 ps and *τ*_cav_ = 1.2 ps. Stimulated emission is taken into account by the Einstein coefficient in the dipole approximation, $B_{21} = \frac{{4\pi ^2}}{{3\hbar ^2}}\left( {\frac{1}{{4\pi \varepsilon _0}}} \right)\left|\langle {\mathrm{\Psi} _2\left| {{{{{{e}}}}}\vec r} \right|\mathrm{\Psi} _1} \rangle\right|^2$, and the spectral energy density, $\rho _0 \cdot N_{{{{\mathrm{ph}}}}} = \frac{{hv_{{{\mathrm{L}}}}}}{{V\Delta v_{{{\mathrm{L}}}}}} \cdot N_{{{{\mathrm{ph}}}}}$. Here, $\Delta v_L = \frac{1}{{2\pi }}\left( {\frac{1}{{\tau _1}} + \frac{1}{{\tau _2}}} \right)$ is the bandwidth of the laser transition at *v*_L_ = 2.2 THz and *V* = *A*_Laser_∙*d* describes the mode volume. The transition dipole moment $\mu _{21} = \langle\mathrm{\Psi} _2\left| {e\vec r} \right|\mathrm{\Psi} _1 \rangle= 8.5\,{{{\mathrm{nm}}}} \cdot e$ with the elementary charge, *e*, is extracted from a band structure simulation of the QCL. The injection of electrons into level 2 is determined by the pump rate *G* and limited by the pre-factor (*N*_e_ − *N*_2_) implementing Pauli blocking by including a maximal electron number in the upper level of *N*_e_ = 0.9∙10^9^.

At delay time *t* = *t*_perturb_ = 0 ps, we increase the photon population using a short, delta-shaped term *β*⋅*δ*(*t* − *t*_perturb_). Here, $\beta = 5.7\cdot{{10}^{7}}$ ${{{{\mathrm{ps}}}}^{-1}}$ describes the strength of the perturbation and is chosen to fit the experimental data and to increase *N*_ph_ by approximately an order of magnitude. The subsequent dynamics of the cavity photon number back to quasi-equilibrium are fitted with a decaying exponential function in order to extract the gain recovery time.

### Microscopic carrier transport model of the QCL

The dephasing time *T*_2_ of the lasing transition in the inset of Fig. 4e has been computed based on self-consistent stationary carrier transport simulations, using a density matrix ensemble Monte Carlo approach^50^. Here, the energy quantization due to electron confinement in growth direction as well as the in-plane electron wave vector are considered, allowing us to include both inter- and intrasubband processes. Space charge effects are considered by coupling the carrier transport simulations to a Schrödinger–Poisson solver, providing the wave functions and eigenenergies of the quantized electron states (Fig. 4d, e). Our carrier transport model accounts for tunneling through the thick injection barrier as well as all relevant scattering mechanisms, such as electron–electron interactions beyond mean-field theory, scattering with acoustic and longitudinal-optical phonons (also accounting for nonequilibrium phonons), and elastic scattering due to impurities and interface roughness. The dephasing time is modeled as 1/*T*_2_ = (1/*T*_u_ + 1/*T*_l_)/2 + γ_p_. Here, *T*_u_ and *T*_l_ denote the upper and lower laser level lifetimes obtained from the corresponding intersubband scattering rates, and γ_p_ is the pure dephasing rate due to intrasubband processes, which is also self-consistently calculated in our simulation approach^50^.

## Supplementary information

Supplementary Information
