# Supplementary material for: Field-resolved high-order sub-cycle nonlinearities in a terahertz semiconductor laser
Source: Light Sci Appl. 2021 Dec 20;10:246. doi: 10.1038/s41377-021-00685-5 (PMC8685277; doi:10.1038/s41377-021-00685-5)
Supplement: Supplementary file 1 — Supplementary Information [file 41377_2021_685_MOESM1_ESM.docx]

## **Supplementary Information for**

## **“Field-resolved high-order sub-cycle nonlinearities in a terahertz semiconductor laser”**

J. Riepl^1^, J. Raab^1^, P. Abajyan,^2^ H. Nong^2^, J. R. Freeman,^3^ L. H. Li,^3^ E. H. Linfield,^3^ A. G. Davies,^3^
A. Wacker^4^, T. Albes^5^, C. Jirauschek^5^, C. Lange^6^, S. S. Dhillon^2,*^ and R. Huber^1^

*^1^Dept. of Physics, University of Regensburg, 93040 Regensburg, Germany*

*^2^Laboratoire de Physique de l’Ecole Normale Supérieure, ENS, Université PSL, CNRS, Sorbonne Université, Université de Paris, 75005 Paris, France
^3^ School of Electronic and Electrical Engineering, University of Leeds, Woodhouse Lane, Leeds LS2 9JT, UK
^4^* *Mathematical Physics and NanoLund, Lund University, Box 118, SE-221 00 Lund, Sweden
^5^ Dept. of Electrical and Computer Engineering, Technical University of Munich, 80333 Munich, Germany
^6^ Dept. of Physics, TU Dortmund University, 44227, Dortmund, Germany
* Email: Sukhdeep.Dhillon@phys.ens.fr*

**Experimental THz waveform**


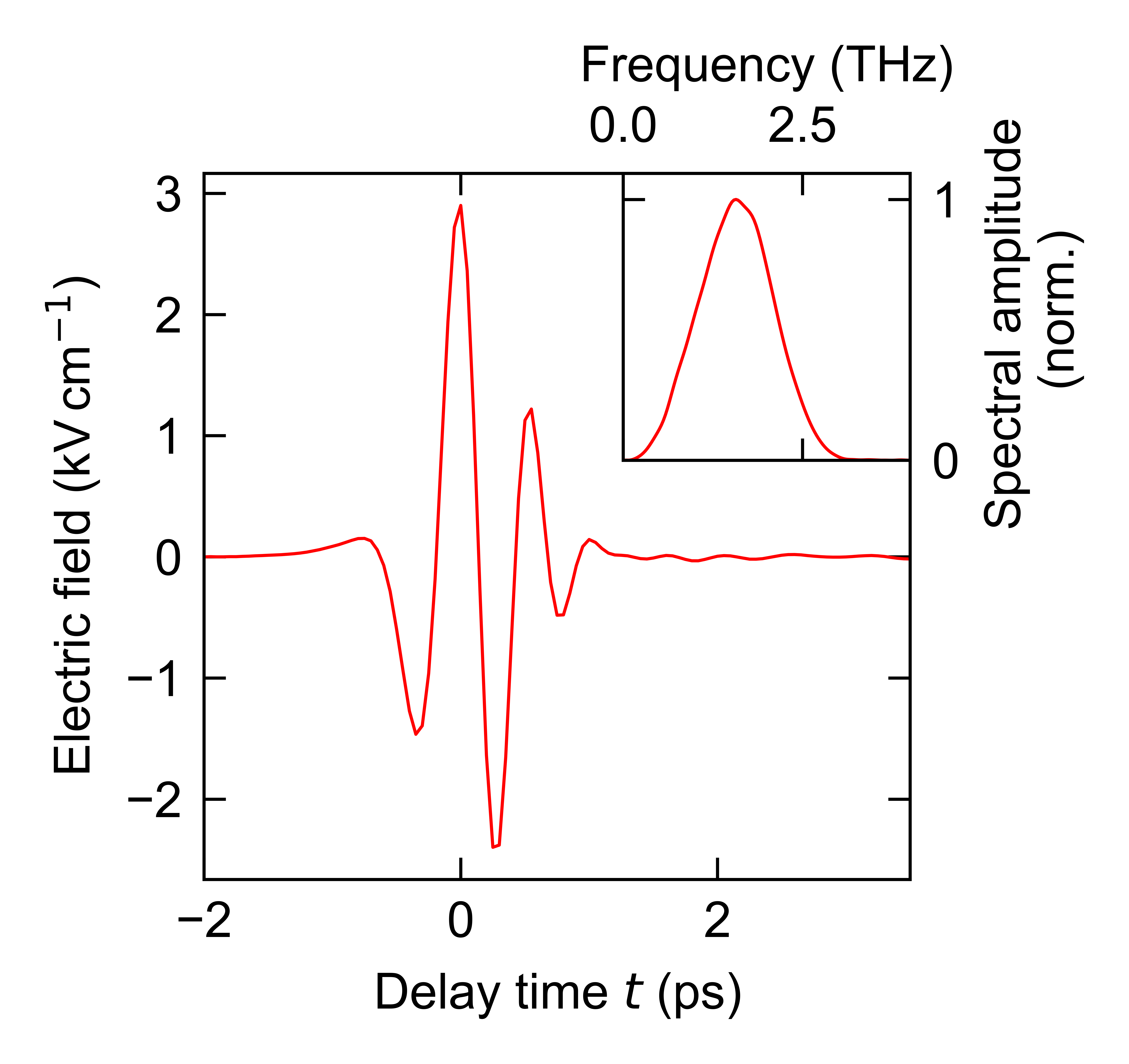


**Figure S1. Experimental THz waveform.** Electric field of THz transients as a function of the delay time t, measured by electro-optic detection. Inset: Corresponding amplitude spectrum.

**Solution of rate equation model for the QCL gain dynamics**

Figure S2 shows representative solutions of the rate equation model (see Materials and Methods) for different pump rates. With increasing pump rate $G$, we clearly observe an increase of the population inversion *n*_2_ - *n*_1_ = $\frac{1}{A_{\mathrm{Laser}}}$ (*N*_2_ - *N*_1_) which is limited by gain clamping as the pump drives the system beyond the laser threshold (Fig. S2a). Here, $N_{1}$ and $N_{2}$ describe the electron number in the lower and upper laser level, connected to the corresponding electron densities $n_{1}$ and $n_{2}$ by the cross-sectional area $A_{\mathrm{Laser}}$ of the laser perpendicular to the growth direction of the QCL. Figure S2b displays the spectral energy density ${\rho_{0}\cdot N}_{\mathrm{ph}}= \frac{hv_{L}}{V\Delta v_{L}}{\cdot N}_{\mathrm{ph}}$ as a function of the delay time *t.* $N_{\mathrm{ph}}$ is the photon number in the cavity, ${\Delta v}_{L}$ is the bandwidth of the laser transition at $v_{L}=2.2 \mathrm{THz}$ and $V=A_{\mathrm{Laser}}\cdot d$ describes the mode volume, including a mode extension in growth direction of $d=50 \mu m$, resulting from the penetration depth of the laser mode into the substrate, and the thickness of the active region. For pump rates above the laser threshold, a finite photon population is formed in the resonator. For better visibility of the temporal evolution of the spectral energy density, the equilibrium spectral energy density ${\rho_{0}\cdot N}_{ph,\infty}\approx{\rho_{0}\cdot N}_{\mathrm{ph}}\left( t=70 \mathrm{ps} \right)$ was subtracted (Fig. S2c).





**Figure S2. a** Population inversion $n_{2}-n_{1}$ as a function of the delay time t for a pump rate far below ($G=0.01$ps^-1^, grey line), below ($G=0.06$ps^-1^, black line), at ($G=0.11$ps^-1^, blue line) and above ($G=0.16$ps^-1^, red line) laser threshold. **b** Spectral energy density ρ_0_·N_ph_ as a function of t. For pump rates below the laser threshold, ρ_0_·N_ph_ tends towards zero. **c** Temporal evolution of the spectral energy density around the time of the perturbation. For illustrative purposes, the equilibrium photon number N_ph,∞_ ≈ N_ph_(t = 70 ps) is subtracted.

**Nonlinear THz dynamics of the unbiased QCL**

Figure S3 shows the normalized two-dimensional amplitude spectrum $\tilde{E}_{\mathrm{NL}}(\nu_{t}, \nu_{\tau})$ of the nonlinear electric field $E_{\mathrm{NL}}(t, \tau)$ (Fig. 2b of the main manuscript) for the unbiased QCL. The dynamics contain oscillations at the transition frequency at ν*_I_* _= 0_ = 1.7 THz, which include pump-probe and four-wave mixing signatures (Fig. S3). Here, six- and eight-wave mixing signals are absent, and the amplitude of the pump-probe and four-wave mixing signals is reduced by more than one order of magnitude with respect to the corresponding nonlinearities recorded in the biased structure (see Fig. 2 of the main manuscript).


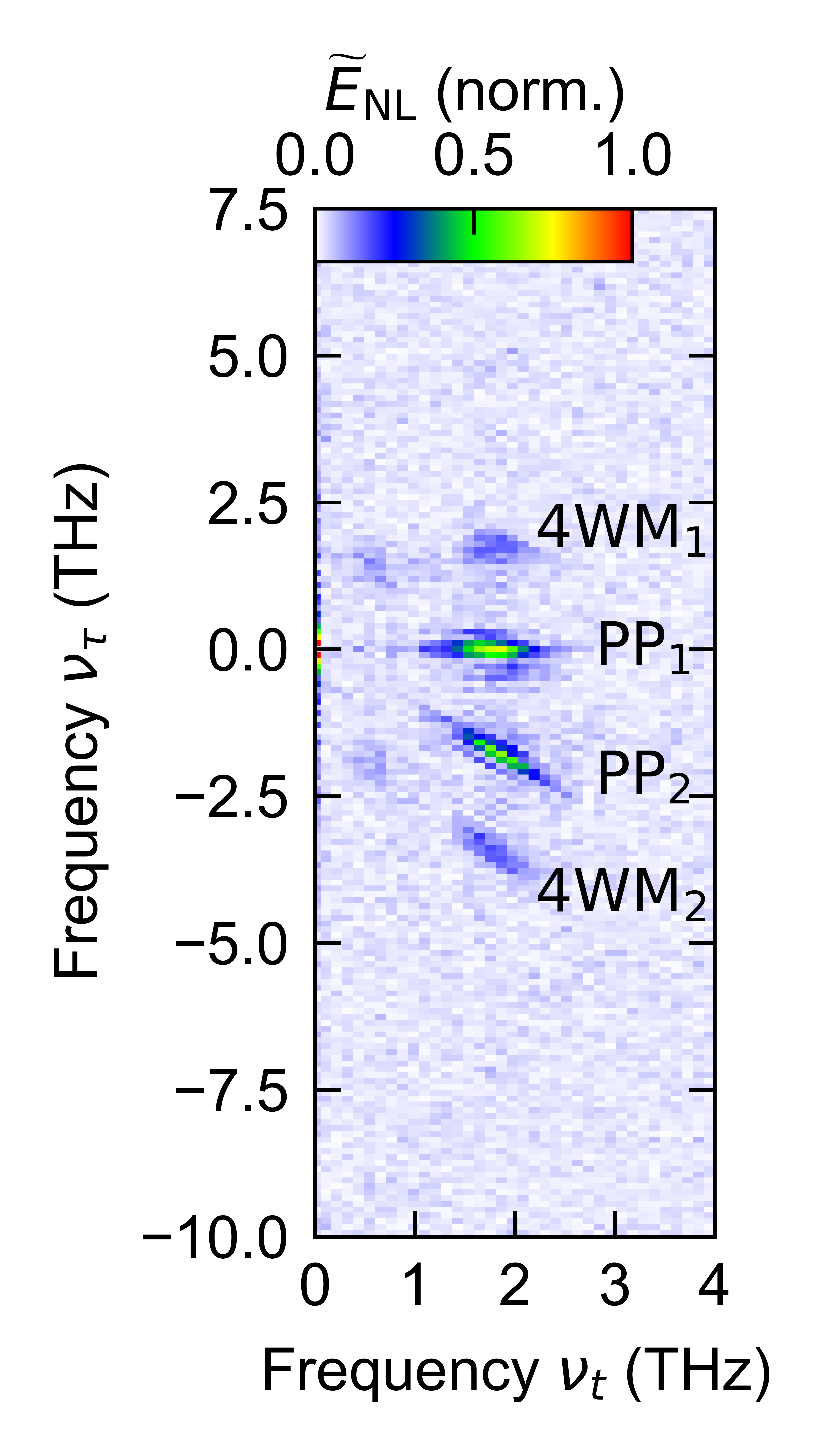


**Figure S3. Field-resolved** **nonlinear** **2D THz spectroscopy of the unbiased QCL.** 2D amplitude spectrum $\tilde{E}_{\mathrm{NL}}(\nu_{t}, \nu_{\tau})$ of the nonlinear response.

**Gain recovery time**

It is possible to extract the gain recovery time $T_{\text{gr}}$ directly from the pump-probe contribution in the time domain data $E_{\mathrm{NL}}(t, \tau)$, since $E_{PP1}$ is the only nonlinear interaction process that does not oscillate as a function of $\tau$. To this end, we reduce the measurement to slices of $E_{\mathrm{NL}}(t, \tau)$ for fixed delay times such as *t* = 1.0 ps (see Fig. S4a, black dashed line) to rapidly determine consistent sets of $T_{\text{gr}}$. The resulting electric field is shown in Fig. S4b as a function of $\tau$. For $\tau$ ≳ 0.7 ps, we apply a fit function of the form:

$E_{\mathrm{NL}}\left( t=1.0 \text{ps}, \tau\right)=A_{1}\exp\left( -\frac{\tau}{T_{\text{gr}}} \right)+A_{\mathrm{osc}}\sin\left( 2\pi\nu_{L}\tau\right)\exp\left( -\frac{\tau}{T_{\mathrm{osc}}^{*}} \right)+c$ (S1)

The first term is only sensitive to the exponential decay of the PP_1_ signal because it is the only contribution in the nonlinear spectrum located at $\nu_{\tau}=0$ (see Fig. 2e of the main manuscript). By contrast, the second term of the fit function oscillates at $\nu_{L}=2.2 \mathrm{THz}$ and, therefore, accounts for contributions from $E_{PP2}$ and $E_{4WM1}$ located at $\nu_{\tau}=\pm2.2 \mathrm{THz}$. The offset c accounts for thermal effects in the QCL cavity on the order of a few percent of the total nonlinear signal.





**Figure S4. Extraction of the gain recovery time from slices of the full nonlinear time-domain signal. a** Total nonlinear signal *E*_NL_(*t*, $\tau$) for a bias current of *I*_B_ = 920 mA. **b** Slice through $E_{\mathrm{NL}}$ as a function of $\tau$ for a fixed delay time of *t* = 1.0 ps. Orange line: numerical fit.

**Estimation of third-order nonlinearity**

In order to estimate the value of the third-order nonlinear susceptibility, $\chi^{\left( 3 \right)}$, we use the wave equation

$\frac{\partial^{2}}{\partial z^{2}}E_{4WM}-\frac{n^{2}}{c^{2}}\frac{\partial^{2}E_{4WM}}{\partial t^{2}}=\frac{1}{\varepsilon_{0}c^{2}}\frac{\partial^{2}P_{4WM}^{\mathrm{NL}}}{\partial t^{2}}$ (S2)

to describe the electric field of the four-wave mixing signals $E_{4WM}\left( z,t \right)=A_{4WM}\left( z,t \right)ⅇ^{ⅈ\left( k_{4WM}z-\omega_{L}t \right)}+c.c.$, oscillating at $\omega_{L}=2\pi v_{L}$. The two incident THz fields $E_{i}\left( z,t \right)=A_{i}\left( z,t \right)ⅇ^{ⅈ\left( k_{i}z-\omega_{L}t \right)}+c.c.$, $\left( i=1,2 \right)$ are included in the source term of the 4WM signal, i.e., the third-order nonlinear polarization:

$P_{4WM}^{\mathrm{NL}}\left( z,t \right)=\varepsilon_{0}d_{\mathrm{eff}}A_{1}A_{1}A_{2}^{*}ⅇ^{ⅈ\left( {(k}_{1}+k_{1}-k_{2})z-{({\omega_{L}+\omega}_{L}-\omega}_{L})t \right)}+c.c.$ (S3)

Inserting these definitions into the wave equation, using the relation $k=\frac{n\omega}{c}$, and the slowly varying amplitude approximation, we obtain the expression

$\frac{\partial A_{4WM}}{\partial z}=\frac{{i\omega_{0}d}_{\mathrm{eff}}}{2nc}A_{1}A_{1}A_{2}^{*}$ (S4)

whereby we have assumed perfect phase matching, $k_{4WM}=k_{1}+k_{1}-k_{2}$.

Next, we integrate Eq. S4 along the cavity from $z=0$ to $z=L=2.9 \mathrm{mm}$ and find the magnitude of $A_{4WM}$:

$\left| A_{4WM} \right|=\frac{{\omega_{L}d}_{\mathrm{eff}}}{2nc}\left| A_{1}A_{1}A_{2}^{*} \right|\cdot L$ (S5)

The size of the relevant tensor element $d_{\mathrm{eff}}$ of $\chi^{\left( 3 \right)}$ is defined by:

$d_{\mathrm{eff}}=\frac{2nc}{\omega_{L}}\frac{\left| A_{4WM} \right|}{\left| A_{1}A_{1}A_{2}^{*} \right|\cdot L}$ (S6)

In our measurements we observe that the 4WM signal accounts for approximately 20% of the total nonlinear signal, which corresponds to a peak field strength of $A_{4WM}\approx0.75 V \mathrm{cm}^{-1}$. In order to estimate $A_{i},$ we recognize that not all spectral components of our incident THz pulses are in resonance with the laser transition. More precisely, owing to the limited bandwidth, at a frequency $\nu_{L}=2.2 \mathrm{THz}$ the spectral amplitude of the incident THz field is reduced by a factor of 2 compared to the amplitude at the centre frequency of the THz pulses (see Fig. S1, inset). For a very conservative approximation of $d_{\mathrm{eff}}$, one could naïvely estimate that, therefore, the peak field strength corresponding to the spectral components resonant with the laser transition is also reduced by the same factor, leading to $A_{1}=A_{2}=0.7 \mathrm{kV}\mathrm{cm}^{-1}$. With the refractive index of GaAs ($n_{\mathrm{GaAs}}=3.6$) we obtain an effective third-order nonlinearity of $d_{\mathrm{eff}}\approx1.2\cdot{10}^{10}\mathrm{pm}^{2} V^{-2}$. We refine this estimate by spectrally filtering the incident THz waveform in the spectral vicinity of the resonance of the QCL corresponding to the bandwidth of the 4WM signals. The resulting strongly reduced effective amplitude$A_{1}$ amounts to less than a tenth of the amplitude of the unfiltered spectrum, which increases the estimate for $d_{\mathrm{eff}}$ by a factor exceeding ${10}^{3}$. Both estimates for $d_{\mathrm{eff}}$ surpasses typical values of conventional THz nonlinearities by orders of magnitude, which we link to a combination of several factors including the large dipole moment of the laser transition (~8.5 nm·*e*, where *e* is the elementary charge) and its resonant enhancement of all four interacting THz waves. Most importantly, the free-running laser provides a unique perk: a negative absorption coefficient resulting from population inversion, which boosts the generated nonlinear wave components rather than diminishing them. This feature makes nonlinearities in operating THz QCLs particularly appealing.

**Density matrix formalism**

We model the dynamics of the QCL with a semiclassical theory based on the Maxwell-Bloch equations, using a self-consistent treatment of the polarization as well as the incident and re-emitted field components beyond the rotating wave approximation.

The gain medium of the QCL is implemented as a two-level system, described by the density matrix $\rho$. The electric field *E*(*t*) is coupled to the gain medium by the Hamiltonian:

$H=\left( \begin{matrix} 0 & \mu_{12}E(t) \\ \mu_{12}E(t) & h\nu_{L} \end{matrix} \right)$ (S7)

Here, *μ*_12_ = 8.5 nm∙*e* and $\nu_{L}$ = 2.2 THz represent the dipole moment and the frequency of the laser transition, respectively. The temporal evolution of the system is described by Equations (4a)-(4c). Initially, the QCL is in an incoherent state (*u* = 0, *v* = 0, $w=w_{0}$), with a slight population inversion of $w_{0}=\rho_{22}^{0}-\rho_{11}^{0}=0.2$. The electric field *E*(*t*) is given by Equation (5), considering the incident THz waveform, $E_{\text{THz}}$, (see Fig. S5) and the electric field reradiated by the electronic polarisation, $\rho_{12}$, scaled by the reradiation parameter $\Gamma=5.7\times{10}^{17}\mathrm{As}m^{-1}$. We use the THz transient displayed in Fig. S5 as incident waveform, $E_{\text{THz}}$. The corresponding amplitude spectrum is centred at 2 THz and has a bandwidth of 1 THz (Fig. S5, inset). The phase of $E_{\text{THz}}$ is taken from the experimental THz waveform, transmitted through the biased QCL (Fig. 1b, red).

In order to reproduce the measured nonlinearities, we perform our calculations analogously to the way the 2D data are taken in the experiment. First, we calculate the interaction of the two-level system with each THz pulse individually (*E*_A_(*t*) and *E*_B_(*t*, $\tau$)). In a second step, the interplay of both THz pulses with the system is simulated (*E*_AB_(*t*, $\tau$)). The nonlinear electric field *E*_NL_(*t*, $\tau$) is then obtained according to:

*E*_NL_(*t*, $\tau$) = *E*_AB_(*t*, $\tau$) – *E*_A_(*t*) – *E*_B_(*t*, $\tau$) (S8)

Figure S6 shows *E*_NL_ for a bias current of $I_{B}=920$mA. *E*_NL_(*t*, $\tau$) is in good agreement with the measured data (Fig. S6a, b), highlighted by slices though *E*_NL_(*t*, $\tau$) at a delay time of *t* = 1 ps (Fig. S6c, black dashed lines in Fig. S6a, b). The accuracy of the simulation is also supported by the very good agreement between theory and experiment of the back-transformed electric fields corresponding to the pump-probe signal $E_{\mathrm{PP}_{1}}$ (Fig. S6d-f) and the four-wave mixing signal $E_{4WM_{1}}$ (Fig. S6g-i). Furthermore, the simulation reveals the underlying temporal evolution of the microscopic dynamics in the free-running QCL, including the population inversion $w=\rho_{22}-\rho_{11}$ and the polarisation $\rho_{12}=u+iv$ as a function of $t$. The resulting trajectory of the Bloch vector strongly depends on the current, as displayed for two currents in Figure 4 and discussed in more detail in the main text.


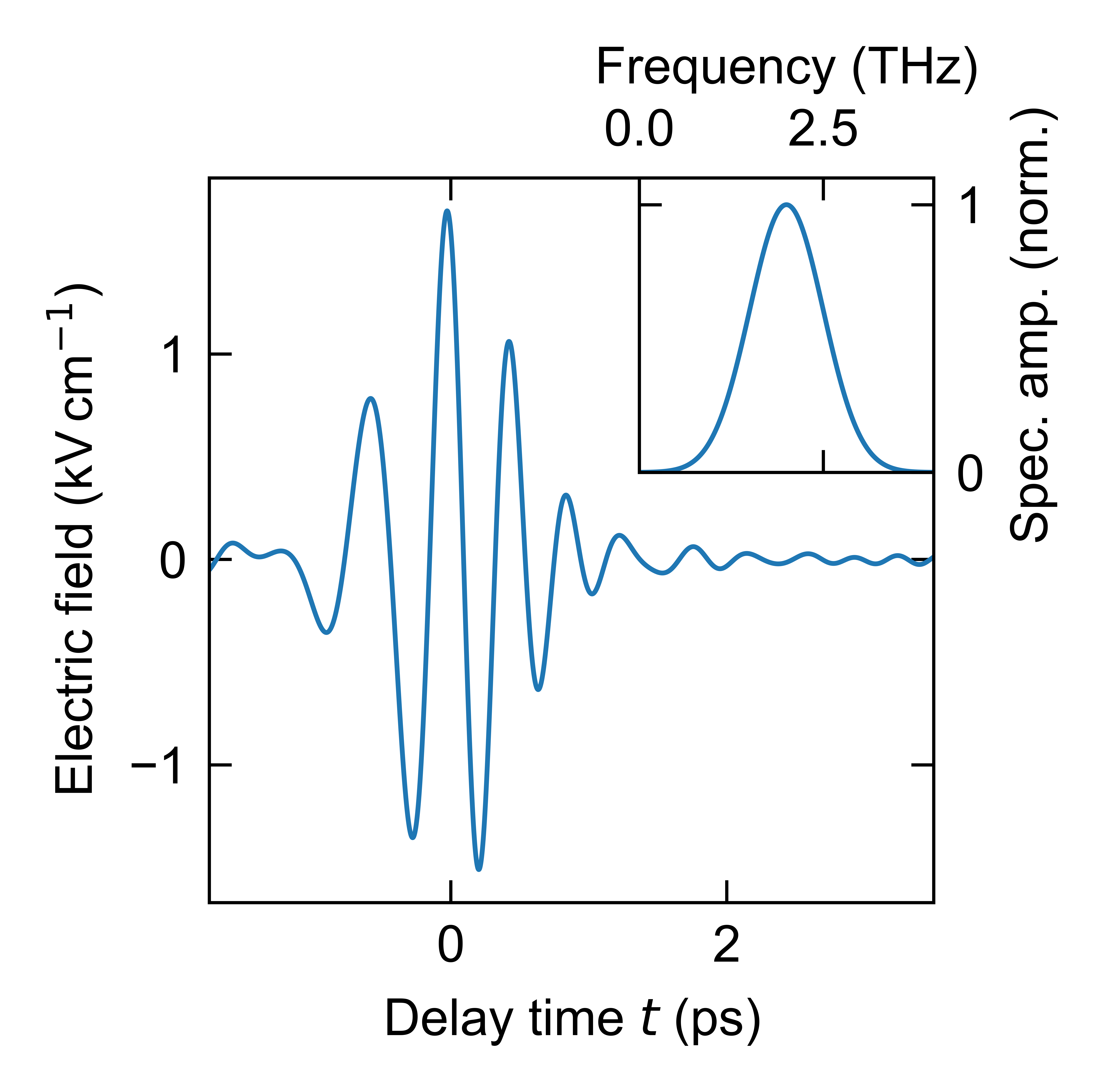


**Figure S5. THz waveform used for simulations.** Inset: Fourier transform of the THz transient used for simulations.


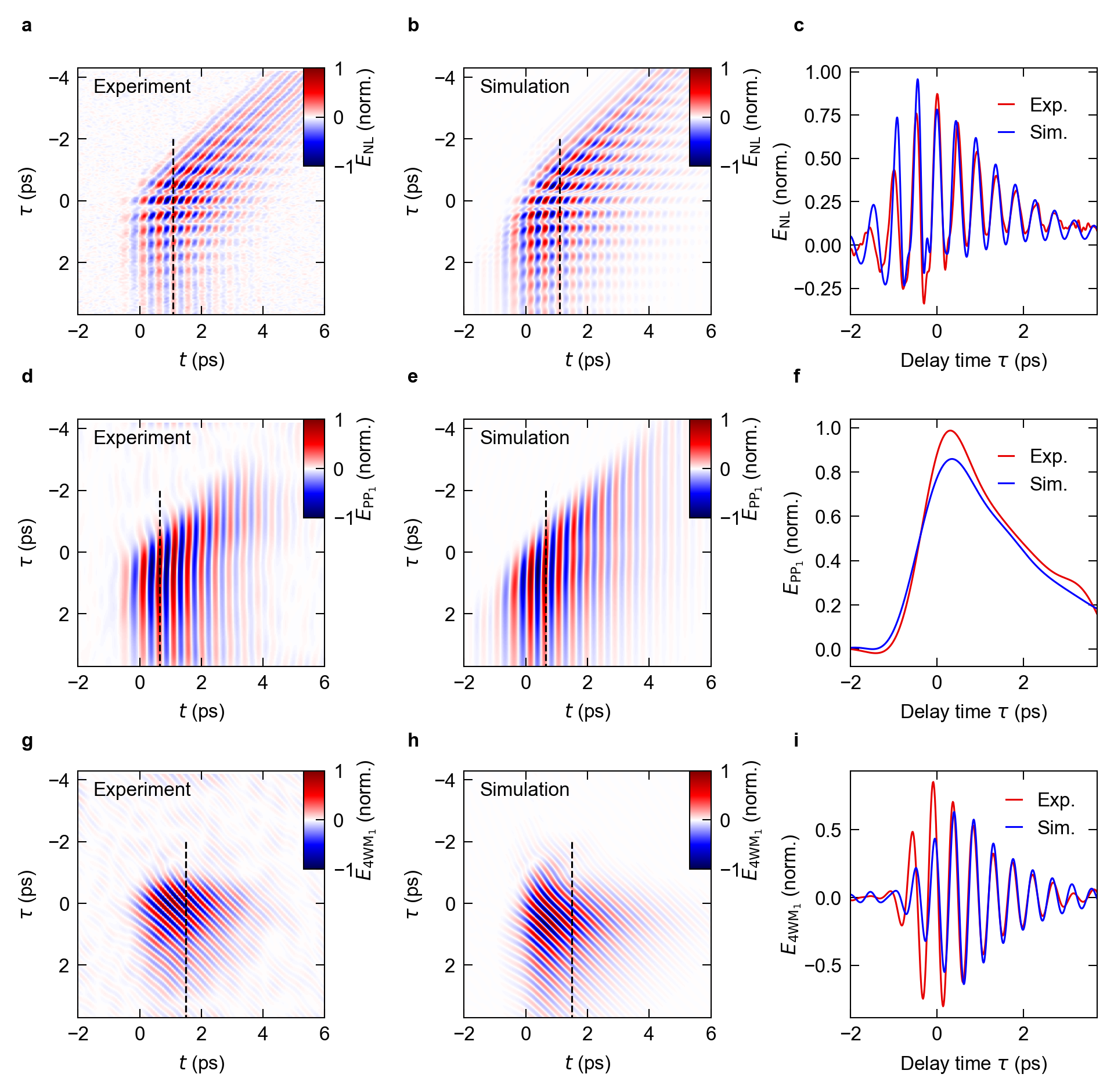


**Figure S6. Density matrix formalism for a free-running QCL. a** Measured electric field *E*_NL_(*t*, $\tau$), emitted by the nonlinear polarization, for a bias current of *I*_B_ = 920 mA as a function of the delay times *t* and $\tau$. **b** Corresponding simulation (see text for details). **c** Slice through *E*_NL_(*t*, $\tau$) at a delay time of *t* = 1 ps (see black dashed lines in (**a**), (**b**)), comparing experiment (red) and theory (blue). **d, e** Back-transformed electric field corresponding to the pump-probe signal $E_{\mathrm{PP}_{1}}$ of measured (**d**) and simulated (**e**) data. **f** Slice through $E_{\mathrm{PP}_{1}}$(*t*, $\tau$) at a delay time *t* = 0.65 ps (see black dashed line in (**d**)**,** (**e**)) comparing experiment (red) and theory (blue). **g, h** Back-transformed electric field corresponding to the four-wave mixing signal $E_{4WM_{1}}$ of measured (**g**) and simulated (**h**) data. **i** Slice through $E_{4WM_{1}}$(*t*, $\tau$) at a delay time *t* = 1.3 ps (see black dashed line in (**g**)**,** (**h**)) comparing experiment (red) and theory (blue).

**Spectral characteristics of the QCL gain**

Figure S7a displays the transmitted THz waveform after a single pass through the biased (red) and unbiased QCL (blue) for a peak field of *E*_p_ = *E*_0_ = 1.4 kV cm^-1^. As discussed in the manuscript, we observe that the field transmitted through the biased laser is approximately 30% higher and shows a clear oscillation for delay times *t* > 1 ps owing to the amplification of the incident THz pulse by stimulated emission in the inverted active medium. A Fourier transform of the two waveforms provides the full spectral characteristics of the QCL gain, encompassing both the spectral amplitude (Fig. S7b) and phase (Fig. S7c). At the transition frequency of the QCL ($v_{L}=2.2 \mathrm{THz}$), the spectral amplitude of the biased device features a clear increase over the unbiased situation. Furthermore, around $v_{L}$, also the phase of the electric field transmitted through the biased QCL deviates from the unbiased case. We more clearly visualize these bias-induced effects in Fig. S7c, which shows the phase difference $\phi_{I_{B}\neq0}-\phi_{I_{B}=0}$ (black) between the two cases as a function of frequency. Biasing induces a change in the phase of up to 0.25π. Most importantly, there is a positive slope in the spectral phase at the laser transition, signifying gain rather than absorption, which would come with a characteristic negative dispersion. In order to compare this result with the case of a halved peak field (*E*_p_ = *E*_0_/2), we analysed the corresponding data in the same way (Fig. S7d-e). Besides more pronounced oscillations in the time domain for delay times *t* > 1 ps and the corresponding doubling of the spectral amplitude at $v_{L}=2.2 \mathrm{THz}$, we also observe a larger phase offset of up to 0.45π. Thus, the complex gain dynamics clearly manifests itself also in the phase spectrum.

*

*

**Figure S7. THz transmission through a QCL. a** Measured THz waveform as a function of the delay time *t*, after transmission through the biased (red curve) and unbiased (blue curve) QCL for an incident peak field of *E*_p_ = *E*_0_ = 1.4 kV cm^-1^. **b** Amplitude spectrum $\tilde{E}(\nu)$ of the waveforms in (**a**). **c** Phase spectrum $\Phi(\nu)$ of the waveforms in (**a**). To illustrate the bias-induced change in the phase spectrum, the difference between the unbiased and the biased phase spectrum $\phi_{I_{B}\neq0}-\phi_{I_{B}=0}$ is shown, in addition (black curve). **d-f** As (**a-c**), but for an incident peak field of *E*_p_ = *E*_0_/2.

**Propagation effects**

In principle, the dispersion of the gain medium reshapes the THz waveform continuously as it propagates along the QCL cavity. As a first estimate of this effect, we compare the THz transient transmitted through vacuum with the transient which exits the QCL cavity after a single pass (Fig. 1b). As a result of the strong gain saturation, the two waveforms remain well comparable. More quantitatively, we reperform our density matrix calculation using the THz waveform focused onto the QCL in the experiment (Fig S8b, inset). This allows us to model the cavity region located directly behind the entrance facet, where propagation effects have not affected the transient yet. The resulting nonlinear response *E*_NL_(*t*, τ) is shown in Fig. S8b. For comparison, a second simulation (Fig. S9) employs a typical THz waveform transmitted through the biased QCL (Fig. S9b, inset), hence including the propagation effects of the gain medium for a single pass through the cavity. Even though we kept the same microscopic parameters in the simulations, the back-transformed PP_1_ and 4WM_1_ signals match the experimental data fairly well for both cases (see Figs. S8 and S9). Propagation effects thus play only a subordinate role for the nonlinear response. Nevertheless, future studies which investigate the QCL dynamics inside the laser cavity with spatial resolution are highly interesting.

**

**

**Figure S8. Density matrix calculation based on experimental waveform. a** Measured electric field *E*_NL_(*t*, $\tau$) emitted by the nonlinear polarization, for a bias current of *I*_B_ = 920 mA as a function of the delay times *t* and $\tau$. **b** Corresponding simulation computed using the experimental THz waveform, shown in the inset. **c** Normalized slice through *E*_NL_(*t*, $\tau$) at a delay time of *t* = 1 ps (see black dashed lines in (**a**), (**b**)), comparing experiment (red) and theory (blue). **d, e** Back-transformed electric field corresponding to the pump-probe signal $E_{\mathrm{PP}_{1}}$ of measured (**d**) and simulated (**e**) data. **f** Normalized slice through $E_{\mathrm{PP}_{1}}$(*t*, $\tau$) at a delay time *t* = 0.65 ps (see black dashed line in (**d**)**,** (**e**)) comparing experiment (red) and theory (blue). **g, h** Back-transformed electric field corresponding to the four-wave mixing signal $E_{4WM_{1}}$ of measured (**g**) and simulated (**h**) data. **i** Slice through $E_{4WM_{1}}$(*t*, $\tau$) at a constant delay time *t* = 1 ps (see black dashed line in (**g**)**,** (**h**)) comparing experiment (red) and theory (blue). The transients are normalized to their respective peak amplitudes.

**

**

**Figure S9. Density matrix calculation based on the THz waveform transmitted through the QCL. a** Measured electric field *E*_NL_(*t*, $\tau$) emitted by the nonlinear polarization, for a bias current of *I*_B_ = 920 mA as a function of the delay times *t* and $\tau$. **b** Corresponding simulation computed using the THz waveform transmitted through the QCL, shown in the inset. **c** Normalized slice through *E*_NL_(*t*, $\tau$) at a delay time of *t* = 1 ps (see black dashed lines in (**a**), (**b**)), comparing experiment (red) and theory (blue). **d, e** Back-transformed electric field corresponding to the pump-probe signal $E_{\mathrm{PP}_{1}}$ of measured (**d**) and simulated (**e**) data. **f** Normalized slice through $E_{\mathrm{PP}_{1}}$(*t*, $\tau$) at a delay time *t* = 0.65 ps (see black dashed line in (**d**)**,** (**e**)) comparing experiment (red) and theory (blue). **g, h** Back-transformed electric field corresponding to the four-wave mixing signal $E_{4WM_{1}}$ of measured (**g**) and simulated (**h**) data. **i** Slice of $E_{4WM_{1}}$(*t*, $\tau$) at a constant delay time *t* = 1 ps (see black dashed line in (**g**)**,** (**h**)) comparing experiment (red) and theory (blue). The transients are normalized to their respective peak amplitudes.
